# The Mental Health Impacts of Fuel Poverty: A Global Scoping Review

**DOI:** 10.3389/ijph.2024.1607459

**Published:** 2024-11-19

**Authors:** S. Khavandi, L. Mccoll, C. Leavey, V. J. McGowan, N. C. Bennett

**Affiliations:** ^1^ Division of Population Health, The University of Manchester, Manchester, United Kingdom; ^2^ Population Health Sciences Institute, Newcastle University, Newcastle upon Tyne, United Kingdom; ^3^ Sheffield Methods Institute, The University of Sheffield, Sheffield, United Kingdom

**Keywords:** fuel poverty, energy poverty, mental health, energy insecurity, fuel insecurity

## Abstract

**Objectives:**

Fuel poverty, defined in this study as a household’s inability to meet basic energy needs, presents a significant challenge. We aimed to map research on the impact of fuel poverty on mental health.

**Methods:**

We searched peer review and grey literature repositories. Studies were considered eligible if they focused on both fuel poverty and mental health.

**Results:**

47 studies were included. 64% were published in the last 3 years. 43% focused on the UK sub-geography, with the next most common being Spain (17%) and China (13%). 55% had a quantitative study design. Conceptualisation and operationalisation of fuel poverty varied across studies and contexts. 58% focused on specific vulnerable populations. 46 studies reported evidence of a detrimental association between fuel poverty and mental health. We broadly summarise the role of fuel poverty on mental health under four headings: economic, social, behavioural and environmental.

**Conclusion:**

We highlight a need for clear and explicit conceptualisation of fuel poverty, in conjunction with consideration of pathways connecting fuel poverty to mental health, to advance the field and facilitate research which can improve population health.

## Introduction

In light of recent global political instability, there has been sustained increases in living costs and a surge in energy prices [1, 2]. This has left many households struggling with the financial costs of meeting their basic needs, including to heat homes, cook, or run everyday appliances [3, 4]. Broadly, an inability to meet these costs is referred to as “fuel poverty” in the academic literature. Three primary drivers have been identified in the literature which push households into fuel poverty: 1) low household income; 2) energy and fuel price increases making bills less affordable; 3) energy efficiency of the home itself [5]. These drivers highlight both the role of wider politics, socioeconomics and the structural qualities of residential dwellings in determining whether a household experiences fuel poverty.

Beyond the primary thermal discomfort resulting from restrictions in the use of domestic heating appliances, fuel poverty also impacts several other facets of life, resulting in detrimental effects on health and wellbeing. Fuel poverty is therefore a social determinant of health. Given global concerns of widening economic and health disparities, and uncertain future energy costs due to ongoing geopolitical instability and the climate crisis, fuel poverty remains a pressing issue. Understanding the complex ways fuel poverty impacts health is paramount to developing targeted interventions and policies to protect and improve population health.

A large body of international research has demonstrated the diverse detrimental effects of fuel poverty on health, morbidity and mortality [6–10]. This body of research has identified many adverse physiological effects of living in fuel poverty, including respiratory conditions such as asthma, bronchitis, infectious diseases [11], exacerbated and increased vulnerability to arthritis [12] and worsened non-communicable diseases such as cardiovascular ailments [13]. The primary pathways through which many of these outcomes are impacted by fuel poverty are via the physical conditions of the home. Colder household temperatures not only impact physical health directly via thermal discomfort, but also contribute to the development of damp and mould which have been further linked to detrimental health outcomes such as in the exacerbation of respiratory diseases [14]. Beyond these physical conditions of the home, behavioural pathways also exist. For example, those which affect households decision making; notably the “heat or eat” factor, whereby households or family members (typically the primary caregiver(s)) forgo meals in order to afford heating, with these dietary changes directly impacting physical health [15]. It is important to acknowledge that the aforementioned pathways detrimentally and disproportionately affect vulnerable population groups such as the elderly, young children, and those with pre-existing health conditions [16].

Whilst prior research has highlighted the clear detrimental physical health consequences of living in fuel poverty, there has to date been limited focus on the mental health impacts of fuel poverty. To our knowledge, no study has yet attempted to comprehensively and systematically review the global literature on the topic. A review of the literature on fuel poverty and health was conducted in 2010 by Liddell and colleagues which included a section on mental health. The authors reviewed studies on the impact of fuel poverty related improvement schemes on mental health and reported significant improvements to mental health [7]. Subsequently, a review specifically focusing on “cold and damp homes” and mental health was conducted [17]. The authors primarily focused on studies on the mental health impacts of heating and insulation improvements. From the nine studies identified, the authors conclude that “cold and damp homes are associated with sub-optimal mental wellbeing” (p.198). Most recently, Champagne and colleagues conducted a scoping review with a European focus on all health outcomes, identifying 15 studies measuring mental health. The authors reported significant detrimental associations between fuel poverty and mental health [13].

This study contributes to the body of research examining the association between fuel poverty and mental health. We aim to review and map the global literature on this association to a) understand how fuel poverty is operationalised as a concept, b) understand the methods used for measuring fuel poverty, and c) understanding the findings of studies in how fuel poverty is related to mental health.

## Methods

This scoping review was conducted according to the Preferred Reporting Items for Systematic Reviews and Meta-Analyses – Extension for Scoping Reviews (PRISMA-ScR) [18]. The protocol has been published elsewhere [19].

### Eligibility Criteria

We applied the Population, Concept, Context (PCC) structure to set our inclusion and exclusion criteria (Table 1) [20]. The population group included people of any age with no additional limitations set. The concept, and focus of this review, was the role of fuel poverty on mental health. Fuel poverty was defined as having difficulties or being unable to afford to pay for gas or electricity to do basic household tasks like cook, turn on the lights, run a fridge, wash, and maintain a healthy indoor temperature. All measures of fuel poverty including self-reported difficulty with fuel and energy bills, quantitative financial measures of expenditure on fuel, and proxy measures such as reporting living in a “cold home” were included. Mental health was defined broadly, encompassing mental wellbeing and common mental disorders. In terms of context, all countries were considered. The context of fuel poverty was the home. Other residence types (such as institutionalised populations) were not included. Studies assessing only fuel availability, fuel type or physical barriers to fuel access were excluded due to incompatibility with our conceptualisation of fuel poverty and incomparability with the majority of the studies identified by the review. We limited inclusion of studies to empirical research and grey literature. Both quantitative and qualitative studies were included as no restrictions on methodological approach were imposed.

**TABLE 1:** Inclusion and exclusion criteria (United Kingdom, 2024).

Inclusion	Exclusion
Peer Reviewed empirical research or grey literature	Conferences proceedings, editorials, letters, comments, erratum, survey note, doctoral thesis, review or does not meet population, concept, context criteria
The publication includes a measure of mental health modelled as an outcome of fuel poverty. In the case of qualitative studies, the publication features specific discussion of mental health impacts of fuel poverty as a substantive theme in the results	The publication employs ecological mental health data or does not discuss substantive results relating to the mental health implications of fuel poverty (in the case of qualitative studies). Studies assessing general wellbeing or life satisfaction will be excluded. Studies assessing the mental health impacts only of interventions will be excluded. Similarly, quantitative studies which analyse the mental health of participants in improvement schemes prior to scheme initiation will be included only where a reference group not experiencing fuel poverty is also included. Studies which do not report any empirical research on fuel poverty and mental health will be excluded
The publication focuses on the home environment in relation to fuel poverty	The publication focuses on any other residential unit

### Search Strategy

We conducted our search on: Medline (Ovid), PubMed Central, APA PsychInfo (Ovid), Web of Science, Scopus, Embase (Ovid), Social Policy Practice (Ovid), Econlit (Ovid) and The Cochrane Library. Grey literature was searched in databases including: OpenGrey, Grey Literature Report, the WHO database, Department for International Development research output database. The search was conducted on the 29th March 2023 with no date restrictions imposed. A second search was conducted on the 12th of February 2024 to capture any further articles. Search strings are available in the appendix.

### Screening, Selection and Charting

All identified studies were screened against our pre-defined eligibility criteria (Table 1). Title and abstract screening were completed by LM and CL. Full text screening was performed by NB and SK. Where there was uncertainty at any stage of the screening process, this was resolved with discussion between authors in line with scoping review methodology [21, 22]. Included studies were extracted by two authors (NB & SK) into a table created by the authors prior to the extraction phase. Extracted data were aggregated and checked for consistency by two authors (NB & SK) and any discrepancies resolved. Themes, definitions, quantitative measures, and results from included studies were extracted. We employed a narrative approach to the synthesis of the data extracted from the included studies in relation to fuel poverty and mental health associations and the pathways involved in the relationship.

### Public and Community Involvement and Engagement

Public contributors were involved throughout the project to help guide the research and to ensure the relevance of the project and its findings to those with lived experience of fuel poverty. We regularly engaged with a steering group of public contributors from the NIHR Applied Research Collaboration for Greater Manchester and held public involvement sessions with members of two community centres (Larkspur and Edberts House) in Newcastle upon Tyne.

## Results

### Search Results

**FIGURE 1:**
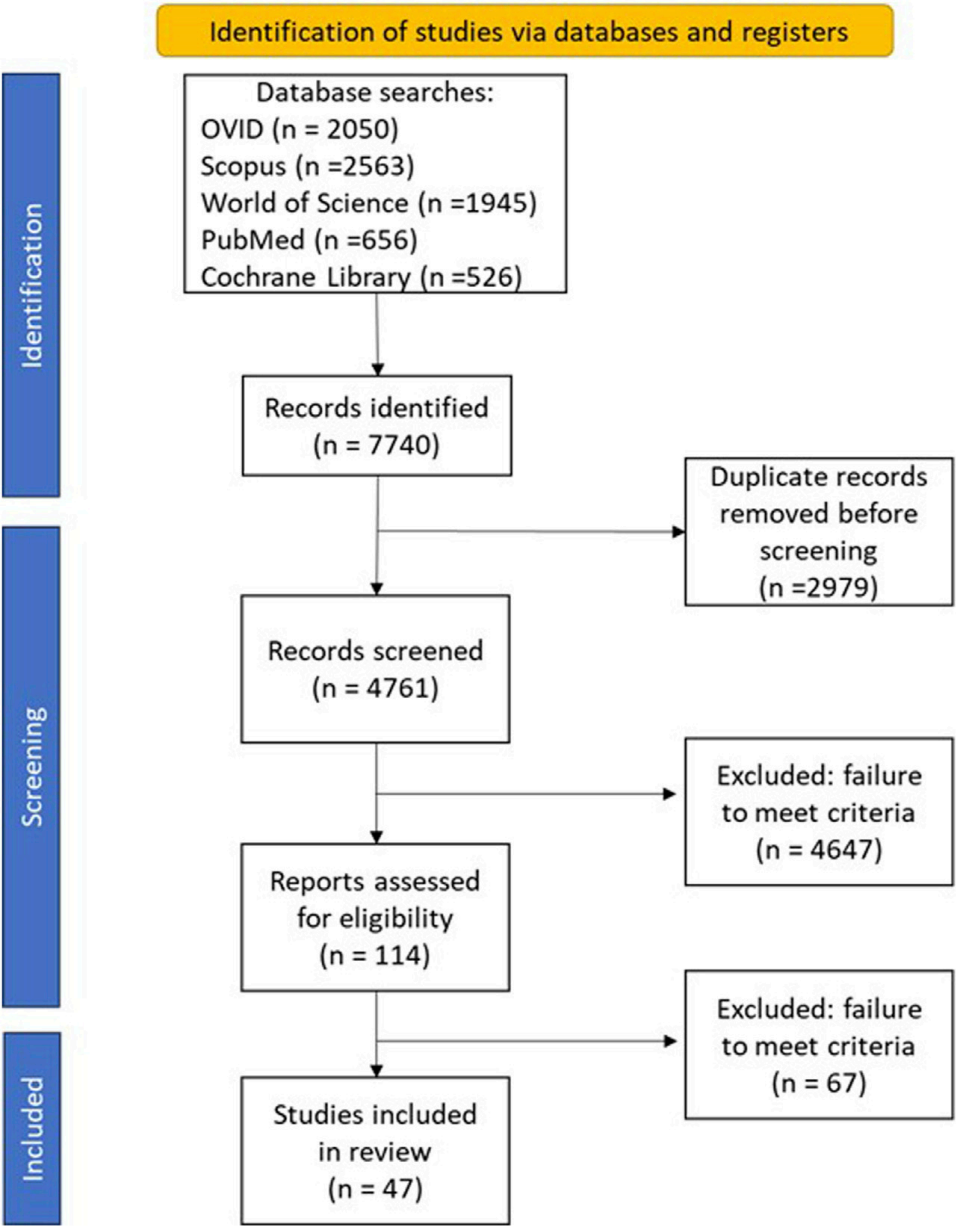
Flowchart for scoping review process adapted from Preferred Reporting Items for Systematic reviews and Meta-Analyses-Scoping Reviews guidelines [18] (United Kingdom, 2024).

Our database search returned a total of 7740 studies, after removal of duplicates 4761 studies went through title and abstract screening of which 114 full-text articles were screened for inclusion (Figure 1). A total of 47 studies were included in the final analysis, 42 from peer-reviewed journals and 5 from grey literature.

### Description of Studies

Included studies were mostly quantitative (n = 26) compared to qualitative (n = 19), with just two studies using a mixed methods approach.

In total, 60% of studies were published within the last 3 years 2023 (n = 3), 2022 (n = 19), 2021 (n = 7); highlighting the topical nature of fuel poverty research. The remaining studies (n = 18) dated over the last two decades back to 2005.

Most included studies were based in the United Kingdom (UK) (n = 20), eight were based in Spain, six in China, four in Australia, three in the United States of America, two in Ireland and one study from each of the following countries: Belgium, Canada, Hungry, New Zealand, North Macedonia, Norway and Poland. Table 2 provides a full breakdown of these data.

**TABLE 2:** Description of included studies (United Kingdom, 2024).

	N	(%)
Date of publication		
2020–2023	30	64
2016–2019	9	19
2011–2015	6	13
2004–2010	2	4
Study geography^a^		
UK	20	43
Spain^b^	8	17
China	6	13
Australia	4	9
United States	3	6
Ireland	2	4
Belgium	1	2
Canada	1	2
New Zealand	1	2
Norway	1	2
Poland^b^	1	2
Hungary^b^	1	2
North Macedonia^b^	1	2
Study design		
Quantitative	26	55
Qualitative	19	40
Mixed methods	2	4
Fuel poverty measure		
Ratio based	7	27
Unmet energy need	5	19
Physical household environment	4	15
Combo	10	38
Physical household environment & Unmet energy need		
Physical household environment & Ratio based	7	70
Ratio based & Unmet energy need	2	20
	1	10
Mental health measure		
CES-D	7	28
Self-reported indication of poor mental health	5	20
GHQ	4	16
SF12 MCS	3	12
SF36 MCS	2	8
SWEMWBS	1	4
PSS	1	4
SDQ	1	4
CIDI SF	1	4

^a^ n.b. some included studies feature more than one country.

^b^ indicates country featured once by the same multi-country study [23].

### Conceptualising Fuel Poverty

Many different definitions of fuel poverty were used among the included studies. Often, the quantitative measure used to capture those in fuel poverty was also operationalised as the definition of the concept. Broadly, the definitions used can be categorised into four types, based on: unmet energy needs (22 studies); a ratio based on energy expenditure and income (five studies); indicative qualities of the physical household environment (three studies); and finally, any combination of the former definitions (five studies). These definitions were variously operationalised as measures taking the form of objective calculations or as self-reported measures. A total of 12 studies provided no clear definition for fuel poverty, with five of these being quantitative studies and seven being qualitative.

### Quantitative Measures of Fuel Poverty

The 26 quantitative studies identified households and individuals in a state of fuel poverty using an applied quantitative measure which could be categorised by the aforementioned conceptual definitions. See Supplementary Table SA1 for a detailed summary of results by study.

Studies applying a quantitative measure most commonly used a single method for measuring fuel poverty (n = 16). A Ratio based definition was the most commonly used of these (n = 7). Of these, six used Boardman’s’ ratio by applying a threshold at which if a households energy expenditure is greater than 10% of their income they can be classed as being in fuel poverty [24], one used Hills’ Low Income High Cost (LIHC) indicator to apply two thresholds (one on each income and cost), at below 60% of median income and at costs higher than median modelled bill [25]. Only one of these studies measured income after tax and housing costs [26]. An unmet energy need definition was second most commonly used (n = 5). Of the studies applying an unmet energy need definition to measure fuel poverty, four used an indicator based on financial difficulties regarding energy bills and one used an indicator based on behaviour changes in usage of energy.

Four studies measured fuel poverty using a physical household environment definition, of which, three used a subjective question asking participants questions regarding the ability to keep their homes warm and one study used an objective measure by taking recordings of internal household temperature.

Seven studies used a combination of physical household environment with an unmet need, two used physical household environments with a ratio and one used unmet need with a ratio measure.

### Quantitative Measures of Mental Health

The majority of quantitative studies used an instrument measure (n = 20) to quantitatively measure mental health. Of these studies, seven used the Centre for Epidemiologic Studies Depression Scale (CES-D), four used the General Health Questionnaire 12 (GHQ-12), three used the Mental Component of the Short Form 12 Health survey (SF12-MCS), two used the Short Form 36 health survey. Additionally, the following measures were each used once by different studies: the Short Warwick-Edinburgh Mental Wellbeing Scale (SWEMWBS), the Strengths and Difficulties Questionnaire (SDQ) (a screening tool for mental health specifically for children and young people), the Perceived Stress Scale (PSS), and the Composite International Diagnostic Interview Short Form (CIDI-SF).

The five remaining quantitative studies did not use validated instruments to capture mental health, and instead used various self-reported indications of having experienced depressive disorders or anxiety.

### Fuel Poverty and Mental Health

Forty-six of the 47 included studies found a detrimental association between fuel poverty and mental health, with one quantitative study reporting no effect [27].

Of the 26 quantitative studies six studies showed heterogeneity in results by different population groups, including age, sex, ethnicity, students in further education, deprivation and parenthood. Three of these studies showed a large negative association between fuel poverty and mental health in elderly populations [11, 28, 29]; two studies reported worse outcomes in parenthood with mothers experiencing higher likelihood of postpartum depression [30, 31]; one study reported negative associations in vulnerable groups experiencing housing insecurity [32]; and one study reported differences between men and women, demonstrating worse outcomes among men [12].

Eleven of the 19 qualitative studies focused on specific population subgroups. Four of these were based on financially vulnerable groups, where households were chosen to partake based on living in social housing, low income and difficulties with fuel bills. All groups reporting worsening mental health due to fuel poverty [33–36]; two studies focused on single parents, both reporting detrimental impacts [37, 38]; one study focused on women and stated detrimental outcomes [39]; another study assessed students with disability and ethnicity of students, showing detrimental outcomes with respect to both groups [40]; and finally one study carried out research on adults with learning difficulties, reporting that their mental health was detrimentally impacted by fuel poverty [41].

### Pathways

**FIGURE 2:**
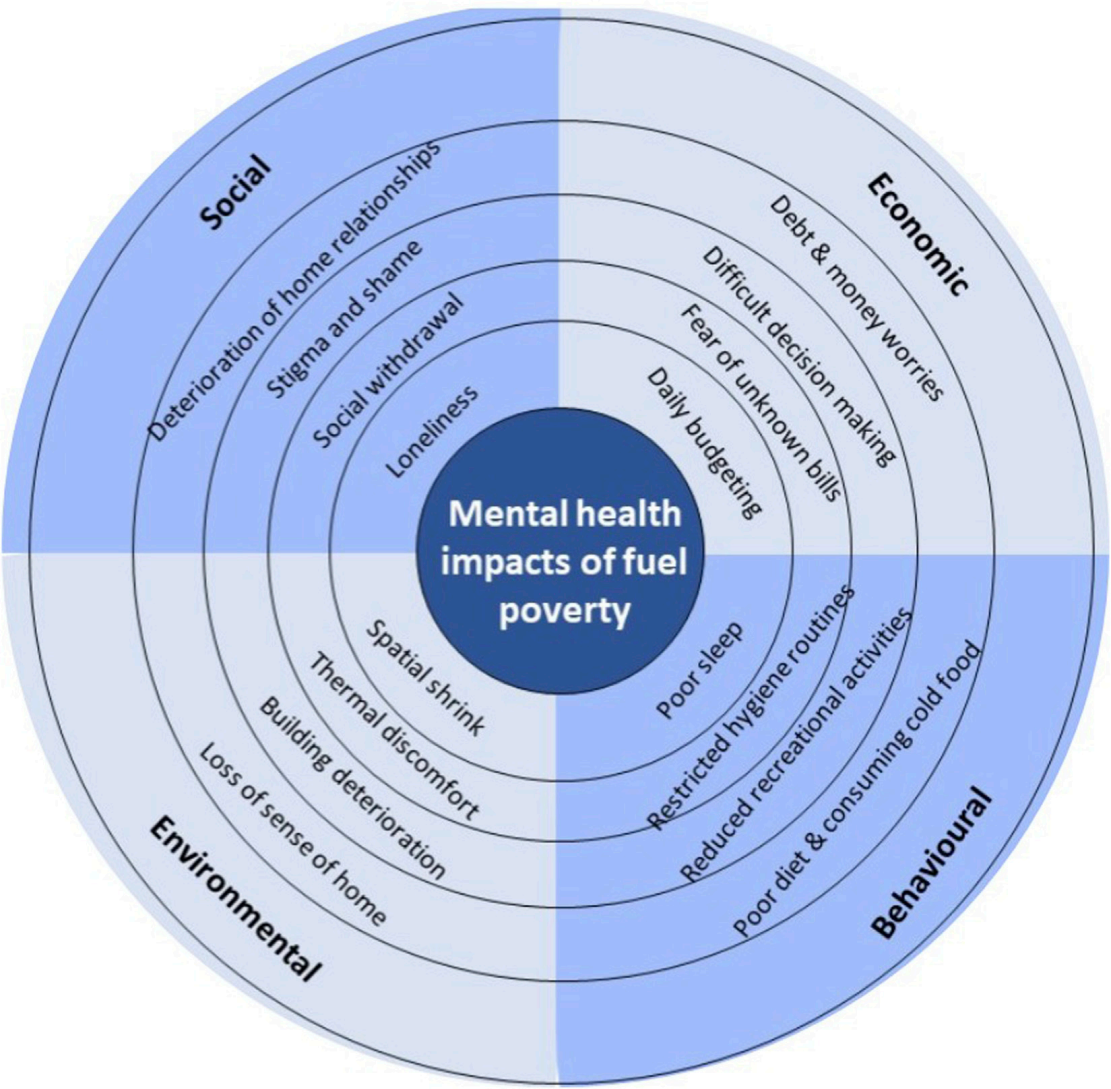
Framework depicting pathways between fuel poverty and mental health (United Kingdom, 2024).

Of the studies included, 37 discussed the pathways thought to operate between fuel poverty and mental health. Where possible pathways were discussed, studies frequently mentioned more than one. Broadly, these pathways can be summarised under the following headings: environmental (n = 20); economic (n = 20); behavioural (n = 10) and social (n = 9) (Figure 2).

We include pathways under the environmental heading where studies described impacts to mental health as a result of the physical environment of the home. Most commonly studies described thermal discomfort (n = 15) as pathway to poor mental health via direct impacts to psychological functioning. For example, Sawyer and colleagues report that the physical health problems cold homes worsen, may also further exacerbate poor mental health [42]. The stress caused by the physical deterioration of the home was also discussed in two studies. The authors also further linked this to the physical health problems that come with mould and damp within households [11]. The concept of “spatial shrink,” whereby the extent of the home lived in is significantly reduced as households decide to heat fewer rooms, was mentioned frequently [30, 36, 42–44]. Authors suggest this not only impacts household relationships via reduced privacy [44], but may also impact the way people interact with their homes, eroding a sense of home and security [30].

Included studies discussing economic pathways described financial insecurity, and often debt, that occurred with fuel poverty (n = 15). Studies often described the challenges households face surrounding the prioritisation of essentials which we term “difficult decision making” (n = 5). Studies commonly referred to the “heat or eat” dilemma, where healthier or heated food options were sacrificed or meals skipped in order to prioritise spending on energy bills [34, 45]. Finally, studies also referred specifically to a fear of receiving bills themselves and these unknown costs (n = 6). All of these were reported to negatively impact mental health with increased stress, worry and anxiety.

Social pathways described hardships encountered as a result of fuel poverty. These included social isolation (n = 6), loneliness (n = 3) and social stigma (n = 6). In studies describing these pathways, fuel poverty was often said to cause withdrawal from social networks and activities due to cost or reluctance to leave the home, and reduced visits from family and friends at home due to housing temperature and condition [42, 43].

Finally, we use the “behavioural” pathways heading to describe significant behaviour changes made by households and individuals in order to lessen the impacts of fuel poverty. These included consciously restricting and carefully planning the use of essential appliances such as washing machines, ovens and central heating in order to optimise the available budget [41, 46] as well as changing hygiene practices, such as forgoing or reducing showering [33]. Studies describing these pathways commonly reported the toll that constant consideration, calculation and modification of these everyday activities has on individuals, as well as the fear of receiving bills which remains. Moreover, studies also frequently cite impacts to sleep (n = 3) and a loss of a sense of home (n = 6).

In general, the qualitative studies more commonly discussed potential explanations for the link between fuel poverty and mental health and explored these themes with participants. Though the quantitative studies often cited potential explanations, these were less thoroughly described and were very rarely empirically investigated in analyses.

## Discussion

This scoping review has described the numerous definitions and measures which have been employed to capture fuel poverty, as well as the range of mental health outcomes which have been studied. We then summarised and described the primary pathways by which the literature suggests fuel poverty impacts mental health.

The review reveals the myriad of ways fuel poverty is both conceptualised and operationalised. We found that studies often failed to appropriately describe their conceptualisation or definition of fuel poverty, instead relying on the quantitative measure chosen to evaluate fuel poverty to describe their understanding of the concept itself. However, research demonstrates that different fuel poverty measures, especially objective versus subjective measures capture different people [47] meaning that a person living in fuel poverty could fall within the definition for one measure, but not another, leading to misclassification biases [13]. Furthermore, length of time spent in fuel poverty is rarely factored into these definitions, despite evidence suggesting that longer durations spent in fuel poverty are worse for health [48]. We argue that this lack of clear conceptualisation and theorisation of what fuel poverty is and how it may be (successfully or not) captured numerically is not beneficial to advancing our understanding of the ways that fuel poverty may impact mental health.

Our review highlighted several groups who are more vulnerable to fuel poverty and to the detrimental effects it can have on mental health, including: the elderly, those with long-term health conditions, families with children, and those with disabilities. Despite the common reference to these groups, few studies, especially quantitative studies, focused on the mental health impacts of fuel poverty on particular sub-groups.

We identified a number of pathways that were suggested to be involved in the negative association between fuel poverty and mental health; these included: environmental, economic, behavioural and social pathways.

Several of these pathways from separate groups were hypothesised to impact mental health through similar overarching mechanisms which are well established in the wider literature. These included a changes to sense of home through a reduced ability to use the home to its fullest capacity from isolating heating to one room and losing the sense of pride in one’s home [49–51]. Another mechanism being the stigma associated with being in fuel poverty [52, 53], and via impacts to physical health such as asthma and respiratory problems being exacerbated by cold home temperatures, these have been shown in a recent review to have negative affects including chronic obstructive pulmonary disease, respiratory viral infections and cardiovascular disease [54].

One previous review by Liddel and Guiney (2015) explicitly reflects on the pathways between “living in a cold damp home” and mental wellbeing in energy efficiency intervention studies. They argue that the effects transmit primarily through an accumulation of stressors which they name as: “…low income, fear of debt, damage to possessions from mould and damp stains, stigma, and social isolation” (p.198). The pathways highlighted by the present study reinforce many identified by this existing review, but further emphasise behavioural pathways (sleep, hygiene, diet and recreational activities), which have largely been overlooked in previous reviews.

### Strengths and Limitations

A strength of our review is its focus on the impact of fuel poverty on mental health, which has to our knowledge, not yet been examined systematically across the global literature base. Moreover, we applied a broad range of terminology, and included literature of both quantitative and qualitative methodologies allowing for a better approach in exploring the pathways between fuel poverty and mental health. This approach has allowed us to provide a robust summary of the state of this growing and increasingly important research field. Furthermore, we were able to describe some of the explanatory factors for the association between fuel poverty and mental health.

However, our review was also subject to limitations. Firstly, as described above, the absence of a commonly understood definition of fuel poverty meant comparability between studies, especially across countries, was challenging. In addition, despite allowing for a global focus, this review predominantly contained European countries with the exception of the United States, Canada, New Zealand, Australia and China. As we took a scoping review approach this study, we did not perform critical appraisal of the included studies and therefore cannot comment on the quality of the studies or the robustness of their results.

### Implications for Future Research on Fuel Poverty and Mental Health

Our findings have several implications for future research. Firstly, clearer conceptualisation of fuel poverty is necessary in future studies in order to elucidate the mechanisms operating between fuel poverty and mental health. Secondly, the value of qualitative research in this field is clear in the contributions which have been made to understanding the relationship to date. Future research may benefit from the adoption of mixed methods approaches in order to advance our understanding of the mechanisms at the population level. Finally, this review identified sub population groups who are more vulnerable to the deleterious impacts of fuel poverty. Further research on the experience of fuel poverty in these vulnerable groups is essential.

### Conclusion

This scoping review mapped the current research on fuel poverty and mental health. We found a lack of conceptual clarity on the term “fuel poverty” and identified a wide range of definitions and measures used to date. Overall, the majority of studies, both qualitative and quantitative identified a detrimental association between fuel poverty and mental health. Explanations for this relationship fell into four broad themes: environmental, economic, social, and behavioural. Though it is widely accepted that certain population groups are more vulnerable to the risks of fuel poverty and mental health impacts, few studies analysed these groups specifically. We argue that future work should focus on better understanding these pathways linking fuel poverty and mental health, particularly how these operate in at-risk groups, in order to identify possible interventions.
