# Supplementary material for: The Mental Health Impacts of Fuel Poverty: A Global Scoping Review
Source: Int J Public Health. 2024 Nov 19;69:1607459. doi: 10.3389/ijph.2024.1607459 (PMC11612648; doi:10.3389/ijph.2024.1607459)
Supplement: Supplementary file 1 [file Table1.docx]

Table A.1. Full table of included studies (United Kingdom, 2024).

| Ref | Study | Year | Country | Peer review/ Grey Literature | Quants/Quals | Mental health measure | Fuel poverty measure | Pathways |
| --- | --- | --- | --- | --- | --- | --- | --- | --- |
| 33 | (Ambrose et al., 2016) | 2016 | UK | Grey | Qual | n/a | n/a | Environmental & Economic |
| 54 | (Bartiaux et al., 2021) | 2021 | Belgium | Peer review | Qual | n/a | n/a | Economic & Social |
| 47 | (Bentley et al., 2023) | 2023 | Australia | Peer review | Quant | SF36 | Unmet energy need | Environmental & Economic & Behavioural |
| 35 | (Bredvold & Inderberg, 2022) | 2022 | Norway | Peer review | Qual | n/a | n/a | Environmental & Economic & Behavioural & Social |
| 55 | (Brown & Vera-Toscano, 2021) | 2021 | Australia | Peer review | Quant | SF36 | Ratio based & Unmet energy need | n/a |
| 56 | (Carrere et al., 2021) | 2021 | Spain | Peer review | Quant | Self-reported indication of poor mental health | Physical household environment & Unmet energy need | Environmental & Economic |
| 26 | (Carrere, Belvis, et al., 2022) | 2022 | Spain | Peer review | Quant | Self-reported indication of poor mental health | Physical household environment | n/a |
| 31 | (Carrere, Vásquez-Vera, et al., 2022) | 2022 | Spain | Peer review | Quant | GHQ | Physical household environment & Unmet energy need | n/a |
| 40 | (Chapman et al., 2022) | 2022 | UK | Peer review | Qual | n/a | n/a | Economic & Behavioural |
| 57 | (Clair & Baker, 2022) | 2022 | UK | Peer review | Quant | GHQ | Physical household environment | Environmental |
| 30 | (Corman et al., 2016) | 2016 | USA | Peer review | Quant | CIDI SF | Physical household environment & Unmet energy need | n/a |
| 58 | (Cotter et al., 2012) | 2012 | Ireland | Peer review | Qual | n/a | n/a | Environmental & Social |
| 59 | (Curl & Kearns, 2017) | 2017 | UK | Peer review | Quant | SF12 | Unmet energy need | Economic |
| 60 | (D’arcy, 2022) | 2022 | UK | Grey | Quant | Self-reported indication of poor mental health | Unmet energy need | Economic |
| 61 | (Davillas et al., 2022) | 2022 | UK | Peer review | Quant | SF12 | Ratio based & Physical household environment | n/a |
| 62 | (De Haro & Koslowski, 2013) | 2013 | UK | Peer review | Qual | n/a | n/a | Environmental |
| 11 | (De Vries & Blane, 2013) | 2013 | UK | Peer review | Quant | CES-D | Ratio based | Environmental & Economic |
| 38 | (Gayoso Heredia et al., 2022) | 2022 | Spain | Peer review | Qual | n/a | n/a | Environmental |
| 63 | (Gilbertson et al., 2006) | 2006 | UK | Peer review | Qual | n/a | n/a | Environmental |
| 64 | (Gilbertson et al., 2012) | 2012 | UK | Peer review | Quant | GHQ | Physical household environment & Unmet energy need | Environmental |
| 42 | (Grey et al., 2017) | 2017 | UK | Peer review | Qual | n/a | n/a | Environmental & Economic |
| 25 | (Harrington et al., 2005) | 2005 | UK | Peer review | Qual | n/a | n/a | Environmental & Economic |
| 65 | (Hernández & Siegel, 2019)) | 2019 | USA | Peer review | Quant | Self-reported indication of poor mental health | Physical household environment & Unmet energy need | Environmental & Social |
| 66 | (Hernández et al., 2016) | 2016 | USA | Peer review | Mixed methods | PSS | n/a | Environmental |
| 67 | (Huebner et al., 2023) | 2023 | UK | Peer review | Quant | Self-reported indication of poor mental health | Unmet energy need | Environmental & Behavioural |
| 36 | (Jacques-Aviñó et al., 2022) | 2022 | Spain | Peer review | Qual | n/a | n/a | Economic |
| 39 | (Kiri et al., 2021) | 2021 | New Zealand | Peer review | Qual | n/a | n/a | Environmental & Behavioural |
| 45 | (Longhurst & Hargreaves, 2019) | 2019 | UK | Peer review | Qual | n/a | n/a | Economic & Behavioural |
| 37 | (McHardy, 2013) | 2013 | UK | Grey | Qual | n/a | n/a | Environmental |
| 29 | (Mohan, 2022) | 2022 | Ireland | Peer review | Quant | CES-D | Physical household environment & Unmet energy need | Environmental & Social |
| 44 | (Nie et al., 2021) | 2021 | China | Grey | Quant | CES-D | Ratio based | Economic |
| 12 | (Oliveras et al., 2020) | 2020 | Spain | Peer review | Quant | GHQ | Physical household environment | Economic |
| 43 | (Oliveras et al., 2021) | 2021 | Spain | Peer review | Quant | SDQ | Physical household environment | Environmental & Economic & Social |
| 68 | (Pellicer-Sifres et al., 2021) | 2021 | Spain, Poland, North Macedonia, Hungary | | Qual | n/a | n/a | Economic & Behavioural |
| 69 | (Porto Valente et al., 2021) | 2022 | Australia | Peer review | Qual | n/a | n/a | Economic & Social |
| 70 | (Riva et al., 2023) | 2023 | Canada | Peer review | Quant | n/a | Physical household environment & Ratio based | n/a |
| 41 | (Sawyer et al., 2022) | 2022 | UK | Peer review | Mixed methods | n/a | n/a | Environmental & Behavioural |
| 71 | (Sharpe et al., 2022) | 2022 | UK | Peer review | Quant | SWEMWBS | Unmet energy need | Environmental & Social |
| 72 | (Stack & Meredith, 2018) | 2018 | UK | Peer review | Qual | n/a | n/a | Behavioural & Economic & Social |
| 73 | (Tu et al., 2022) | 2022 | UK | Peer review | Quant | SF12 | Physical household environment & Unmet energy need | n/a |
| 32 | (Waitt & Harada, 2019) | 2019 | Australia | Peer review | Qual | n/a | n/a | Economic & Behavioural |
| 74 | (Wang et al., 2022) | 2022 | China | Peer review | Quant | CES-D | Ratio based | n/a |
| 34 | (Williams et al., 2015) | 2015 | UK | Grey | Qual | n/a | n/a | Economic |
| 27 | (X. Li et al., 2022) | 2022 | China | Peer review | Quant | CES-D | Ratio based | n/a |
| 75 | (Xu et al., 2022) | 2022 | China | Peer review | Quant | CES-D | Ratio based | Economic |
| 28 | (Y. Li et al., 2022) | 2022 | China | Peer review | Quant | n/a | Ratio based | Environmental |
| 76 | (Zhang et al., 2022) | 2022 | China | Peer review | Quant | CES-D | Ratio based | n/a |
